# Does the role of family meetings important at the end-of-life? A retrospective national study in Taiwan

**DOI:** 10.1186/s12904-024-01613-1

**Published:** 2024-12-20

**Authors:** Tsung-Hsien Yu, Chung-Jen Wei, Wei-Wen Wu, Frank Leigh Lu

**Affiliations:** 1 Department of Health Care Management, National Taipei University of Nursing and Health Sciences, Taipei, Taiwan; 2 Department of Public Health, Fu-Jen Catholic University, New Taipei, Taiwan; 3 School of Nursing, College of Medicine, National Taiwan University, Taipei, Taiwan; 4 Department of Nursing, National Taiwan University Hospital, Taipei, Taiwan; 5 Department of Pediatrics, National Taiwan University Children’s Hospital, Taipei, Taiwan; 6 School of Medicine, National Taiwan University, No. 1 Jen-Ai Road, Section 1, Taipei, 100 Taiwan

**Keywords:** Family meeting, Palliative care, Type of end-of-life care, Initiation time

## Abstract

**Background:**

Numerous studies have pointed out the benefits of family meetings, but it is unclear who uses family meetings and what the effects are on use of the end-of-life care.

**Aim:**

The purposes of this study were to explore which characteristics are associated with the use of the family meeting and what effects the family meeting has on end-of-life care.

**Design:**

A retrospective observational study using 2012–2017 data from Taiwan’s National Health Insurance claims database, cancer registry, and death registry.

**Setting/participants:**

People who died between 2013 and 2017 in Taiwan as the study population. The deceased people whose information on gender, marital status, or insured classification that was unknown were excluded from this study.

**Results:**

A total of 792,947 people were included. All demographic characteristics were associated with the use of family meetings, and all demographic characteristics (except for gender and residence area) and hospital characteristics were associated with the initiation time of family meetings. We also found use of family meetings increased the use of hospice care (OR:4.949) and decreased the use of CPR (OR:0.208) at the end of life, initiation time was also associated with the hospice and CPR, but the effects were varied.

**Conclusion:**

This study demonstrated that family meetings affected the care at end-of-life. Although the utilization of family meeting was increased by year, but also variation still existed among demographic and health characteristics, how to promote it is the next concern in the future.

## Introduction

Family meeting or family conference is considered to be an effective strategy to facilitate communication between the patient, family, and clinical teams [1, 2]. Through the family meeting, patients, family members, and clinical team members can discuss the patient’s condition and prognosis, share information regarding the patient’s preferences, and align the goals of care with the patient’s and family’s values and emotional needs [1, 3, 4]. There were studies that have found that family meetings are associated with reduced health care utilization [5] and better quality of the dying experience [6]. Through the information obtained during a family meeting [4], family members can adjust and prepare themselves for their family’s loss. This can reduce psychological distress and disorders (e.g. PTSD, anxiety, and depression) [6–8]. The family meeting could therefore be considered to be a catalyst for hospice and palliative care [9]. 

Through family meetings, health care providers, patients, and family caregivers may clarify their values, provide information, determine care preferences, and identify sources of illness-related distress and burden [10]. Existing studies have found that family meetings can reduce psychological distress and help meet unmet needs among patients and families [11, 12]. However, the study on the utilization of family meeting is rare, through literature review, we only found two studies which were conducted in Taiwan, and both of them were using the data of single hospital, and both of them also selected specific patients as the study population. They found the utilization rate of family meeting was ranged 22-36% [13, 14]. Although current studies offered a snapshot, but the generalizability of their findings is still unknown. Furthermore, the effects of family meeting on the preference of end-of-life care is unknown too. It would be worth to explore who tends to use family meeting at the end-of-life, and whether the family meeting is associated with the preference of end-of-life care [10, 12].

Taiwan’s National Health Insurance (NHI) began reimbursing hospice care services in 1998 and started covering consultation fees for family meetings at the end of 2011. These consultation fees are applicable for patients in the final stages of life. Physicians are expected to conduct these consultations, which should last over an hour, involving both the patients and their family members. Additionally, written minutes of each meeting are required. The content of the family meeting should address the following issues: (1) Current clinical condition and prognosis; (2) Treatment plan and goals; (3) Feasibility of receiving hospice care; (4) Do Not Resuscitate (DNR) orders; (5) Withholding or withdrawing life-sustaining treatment; (6) Advanced directives. The National Health Insurance Administration (NHIA) also regulates that each hospital can only reimburse the consultation fee for family meetings once per patient initially, yielding 1,500 points per patient (with a dynamic point value, typically less than 0.033 USD). After three years of reimbursement, the NHIA lifted the restriction allowing each patient to apply for the consultation fee twice per hospital visit. Concurrently, the points for the consultation fee increased from 1,500 points per meeting to 2,250 points per meeting.

The claims data from the NHI provide researchers with a valuable opportunity to address these questions. This study aims to explore the characteristics associated with the use of family meetings and to examine the impact of these meetings on end-of-life care preferences.

## Methods

### Study design

This was a retrospective, cross-sectional, population-based study.

### Data source and study population

For this study, we used 2012–2017 data from the Taiwan Death Registry, the Taiwan Cancer Registry, and Taiwan’s National Health Insurance (NHI) claims database. The Taiwan Death Registry was used to identified the study population; the Taiwan Cancer Registry to identify if a deceased person had had cancer or not; and the NHI claims data to identify any use of a family meeting and/or hospice palliative care before the person’s death.

To reduce the potential effect of low and unstable utilization of family meeting reimbursements in the initial years of implementation, we selected the people who died between 2013 and 2017 as the study population. We identified their death events and causes of death by consulting the National Death Registry, then accessed their NHI claims data and the Taiwan Cancer Registry to retrieve medical records. NHI claims data was used to identify whether the deceased person had received hospice palliative care in the previous 12 months, and whether any family meetings were held before that person’s death. We also used NHI claims data to retrieve demographic characteristics. The Taiwan Cancer Registry data provided each person’s cancer-related information. The deceased people whose information on gender, marital status, or insured classification that was unknown were excluded from this study.

### Variables of interest

#### Type of end-of-life care

We selected the use of hospice care and the use of CPR at the end of life to illustrate different approaches to end-of-life care. Hospice care represents a model that prioritizes improving the quality of life and maintaining the patient’s dignity rather than prolonging life. In contrast, CPR is associated with a care model focused on extending a patient’s life.

##### Use of hospice care

A person who received any type of hospice care (including hospital-based, shared-care model, home-based, or community-based hospice care services) at least once in the last 12 months before death was classified as using hospice care.

##### Use of CPR at end of life

A person who received cardiopulmonary resuscitation (CPR) at least once in the last 3 days before death was classified as using CPR at the end of life.

#### Utilization of family meeting

Our first observation was whether a study population had held any family meeting or not (use of family meeting). If they did use a family meeting, we also observed the initiation time of the first family meeting. The initiation time was defined as the difference between the date of the first family meeting and the date of death. Since the data distribution of the initiation time of the first family meeting were skewed, for ease of interpretation and clinical application, log transformation and categorization were applied for purpose 1 and purpose 2, respectively. When categorization was applied, the initiation time of family meeting was categorized into: less than 15 days, 16–30 days, 31–45 days, 46-60days, 61–90 days, 91–180 days and more than 181 days.

#### Covariates

We referred the Andersen’s healthcare utilization model to gather our covariates. This model predicts healthcare utilization based on three components: predisposing, enabling, and need. The predisposing component refers to characteristics that increase the likelihood of individuals using healthcare services. This includes demographic factors such as gender and age, as well as social factors like religion and education level. In our study, which utilized the Taiwan Death Registry, the Taiwan Cancer Registry, and Taiwan’s National Health Insurance (NHI) claims database, we were only able to collect age, gender, and marital status to represent this predisposing component.

The enabling component refers to an individual’s ability to access healthcare services. This encompasses personal resources such as income and insurance, as well as regional resources like the level of urbanization of one’s residence, the types of healthcare services available, and the convenience of accessing healthcare. Therefore, we looked at poverty status, urbanization level of residence, and the characteristics of the site of the first family meeting to describe the enabling component. Lastly, health status is the most commonly used indicator for the need component, and we selected the individual’s medical condition as our measure for this component. Details were following:

Predisposing component.

##### Age at death

The study population was classified into seven stratums: 6 years old or younger, 7–12 years, 13–18 years, 19–40 years, 41–65 years, 66–85 years, or above 86 years old.

##### Marital status

Information from the Taiwan Death Registry was used to identify the study population’s marital status, which was classified as single, married, divorced, or widowed.

### Enabling component

#### Poverty

Study population’s insurance identification records from the National Health Insurance Research Database were used to determine poverty status. The NHI program classifies insured people into six insured classifications according to occupation. Households below Taiwan’s designated poverty line belong to classification 5, and this classification was used as a criterion for poverty.

#### Urbanization

Urbanization level was defined based on the study population’s residential area. All 365 townships in Taiwan are classified into seven clusters based on the following indicators: population density (people/km^2^), proportion of people with a college degree or higher, proportion of people over 65 years old, proportion of people who are agricultural workers, and number of physicians per 100,000 people. For this study, residential areas located in clusters 1–3 were categorized as urban, and clusters 4–7 as rural [15]. 

However, Taiwan’s NHI social insurance scheme is based on occupation, and employees of large enterprises might be enrolled using the address of their company’s headquarters rather than their actual residential address. We assumed each person’s actual residential location to be the location where they registered the most outpatient and pharmacy visits. We then recognized the location of each clinic and pharmacy as either urban or rural [16], according to the definition of urbanization published by Taiwan’s National Health Research Institutes.

Characteristics of the site of first family meeting held.

Accreditation level and ownership were used to describe the characteristics of the site of first family meeting held. All family meetings were held in healthcare organizations (including hospitals and clinics) in Taiwan, hospitals in Taiwan are accredited as medical centers, regional hospitals, or community hospitals. Therefore, we classified the accreditation level of the site of the first family meeting held as medical centers, regional hospitals, community hospitals, or clinics. Since only a few family meetings were held in clinics, therefore, we combined clinics and community hospitals. As for the ownership of the site of the first family meeting hold, hospitals and clinics in Taiwan could be owned by the government, not-for-profit organizations, and individuals. Therefore, the ownership was classified as public, not-for-profit, or private.

### Need component

#### Medical condition

We classified the study population into three groups based on medical condition: history of cancer, history of major illness, or none of the above. First we used the Taiwan Cancer Registry to identify whether someone had had cancer or not. For those with no history of cancer, we used the NHI registry of major illness to identify if they had experienced another major illness (e.g., end-stage kidney disease, inherited metabolic disorders, other major illness) or not. The rest of the study population was classified as none of the above.

### Statistical analysis

All statistical analyses were performed using SAS (version 9.3, SAS Institute Inc., Cary, NC, USA). In statistical testing, a two-sided *p* value ≤ 0.05 was considered statistically significant. The distributional properties of continuous variables were expressed by mean ± standard deviation (*SD*), and categorical variables were presented by frequency and percentage. In bivariate analysis, potential predictors of dependent variables were examined using the chi-square test, the two-sample *t*-test, or ANOVA as appropriate, and Scheffé’s test for post hoc comparison. Logistic and multinominal logistic regression were used for multivariable analysis as appropriate.

## Results

A total of 827,692 were died during the study period, the deceased people whose information on gender (*n* = 6,811), marital status (*n* = 9,572), or insured classification (*n* = 18,362) that was unknown were excluded from this study. Finally, 792,947 were retained for further analysis. Table 1 delineates the characteristics of the population included in this study. Most of them were 40 years of age or older, 59.67% were male, and around 82% were married or widowed. Three-quarters (77.89%) of the study population lived in urban areas; a small percentage (6.69%, *n* = 53,060) were identified as living in poverty. Regarding health characteristics, 228,597 (28.83%) had a history of cancer, 190,749(24.06%) had non-cancer-related major illnesses, and others did not have any major illness. A very small percentage (1.55%, *n* = 12,268) had received CPR in the year before death. As for the utilization of family meetings, we find 71,943 (9.07%) had ever held a family meeting. Most of these meetings were held in regional hospitals (32,146, 44.68%) and medical hospitals (28,269, 39.29%), others were held in community hospitals and even clinics. In addition, most of these meetings were held in not-for-profit and public hospitals. The findings also reveal that the average time for the first family meeting was 86.49 days. 27.62% initiated within 2 weeks, 19.77% initiated within 3 to 4 weeks, 19.84% in the second month before death, and 9.02% in the third month. Last,19.77% of the study population had received hospice care, and 13.84% used CPR within 3 days before they died.

**Table 1:** Descriptive analysis of the study population(*N* = 792,947)

Variables	*n* (%)
Year, *n* (%)	
2013	143,895(18.15)
2014	157,326(19.84)
2015	157,919(19.92)
2016	166,839(21.04)
2017	166,968(21.06)
**Demographic characteristics**	
Age of death (years) *n* (%)	
≤ 6	1,278(0.16)
7–12	719(0.09)
13–18	2,104(0.27)
19–40	30,109(3.80)
41–65	206,759(26.07)
66–85	364,624(45.98)
≥ 86	187,354(23.63)
Gender, *n* (%)	
Male	473,162(59.67)
Female	319,785(40.33)
Marital status, *n* (%)	
Single	74,187(9.36)
Married	405,888(51.19)
Divorced	65,824(8.30)
Widowed	247,048(31.16)
Poverty, *n* (%)	
Yes	53,060(6.69)
No	739,887(93.31)
Urbanization, *n* (%)	
Urban	617,614(77.89)
Rural	175,333(22.11)
Medical condition, *n* (%)	
Cancer	228,597(28.83)
Other major illness	190,749(24.06)
None of the above	373,601(47.12)
**Use of family meeting**	
Use of family meeting, *n* (%)	
Yes	71,943(9.07)
No	721,004(90.93)
Place of first family meeting, *n* (%)	
Public Hospitals	28,267(39.29)
Not-for-profit hospitals	35,275(49.03)
Private Hospitals	8,401(11.68)
Level, *n* (%)	
Medical center	28,269(39.29)
Regional hospital	32,146(44.68)
Community hospital	11,517(16.01)
Clinics	11(0.02)
Initiation of 1st family meeting (days), *mean(S.D)*	86.49(151.37)
1–15, *n* (%)	19,869(27.62)
16–30, *n* (%)	14,224(19.77)
31–45, *n* (%)	8,671(12.05)
46–60, *n* (%)	5,601(7.79)
61–90, *n* (%)	6,492(9.02)
91–180, *n* (%)	7,896(10.98)
Above 181, *n* (%)	9,190(12.77)
**End-of-life care**	
Use of hospice palliative care, *n* (%)	
Yes	156,757 (19.77)
No	636,190 (80.23)
Use of CPR (in the three days before death), *n* (%)	
Yes	109,765 (13.84)
No	683,182 (86.16)

**Fig. 1:**
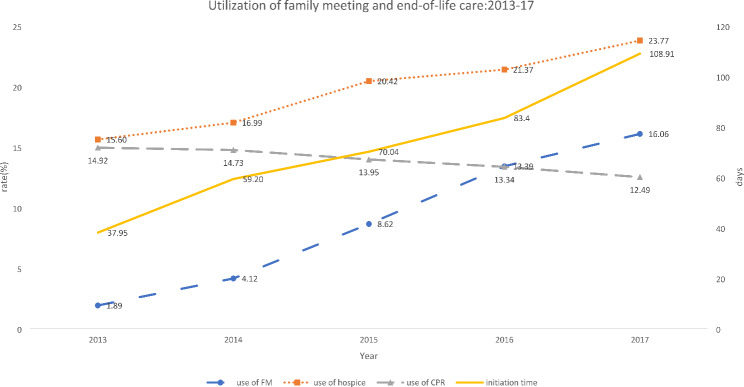
the utilization trend of family meeting and end-of-life care:2013–2017

Figure 1 shows the trend of the utilization of family meetings and end-of-life care during the study period. We found the rate of the use of family increased from 1.89 in 2013 to 16.06% in 2017. Meanwhile, the utilization of end-of-life care was changed as well. With the growth of the use of family meetings, the utilization of hospice care increased from 15.60 to 23.77 and the utilization of CPR decreased from 14.92 to 12.49. in addition, the initiation time of the first family meeting was also increased over time as well. It grew by 2.87 times (from 37.95 days to 108.91 days).

Table 2 depicts the difference in the utilization of family meetings among characteristics. The results show that people who were aged above 40 years old, female, not single, lived in urban areas, and had cancer or major illness had had higher rate of the use of family meetings. People who use family meetings had a higher rate of using hospice (53.46%) and a lower rate of using CPR (2.53%) than their peers. In terms of when the first family meeting was initiated, we found that patients over the age of 66, those who were widowed, poor, or suffering from major illnesses tended to start family meetings earlier. Additionally, family meetings held earlier in private and community hospitals. Our analysis also revealed that patients who did not utilizing hospice care and those opted for CPR were more likely to initiate family meetings at an earlier stage.

**Table 2:** Results of bivariate analysis

	Use of family meeting^1^			Initiation time		
	n	%	p-value	mean	S.D	p-value
Age (years)			***			***
(1) ≤ 6	50	3.91		101.78	118.65	(7)> (6)-(4)
(2) 7–12	52	7.23		67.21	90.75	(6)> (5)-(4)
(3) 13–18	110	5.23		83.91	207.10	
(4) 19–40	1,948	6.47		69.79	128.66	
(5) 41–65	21,216	10.26		72.70	125.88	
(6) 66–85	33,463	9.18		86.69	149.18	
(7) ≥ 86	15,104	8.06		107.61	185.22	
Gender			*			***
Male	42,678	9.02		84.29	146.16	
Female	29,265	9.15		89.71	158.61	
Marital status			***			***
(1) Single	5,414	7.30		84.63	152.02	(4)> (1)-(3)
(2) Married	40,098	9.88		81.14	140.41	
(3) Divorced	5,959	9.05		79.39	130.56	
(4) Widowed	20,472	8.29		99.53	174.9	
Poverty						***
Yes	4,765	8.98		112.71	184.81	
No	67,178	9.08		84.63	148.54	
Urbanization			***			
Urban	59,173	9.58		85.37	143.8	
Rural	12,770	7.28		86.73	152.95	
Medical condition			***			***
(1) Cancer	34,856	15.25		70.02	112.33	(2)> (1),(3)
(2) Major illness	20,888	10.95		125.54	195.16	
(3) None of the above	16,199	4.34		71.6	150.74	
Use of hospice care			***			***
Yes	38,387	53.36		69.54	109.02	
No	33,556	46.64		105.88	186.6	
Use of CPR			***			
Yes	1,819	2.53		119.11	198.09	
No	70,124	97.47		85.65	149.87	
Site of first family meeting						***
(1) Public Hospitals	--	--		82.85	142.76	(3)> (1),(2)
(2) Not-for-profit hospitals	--	--		78.52	131.73	(1)> (2)
(3) Private Hospitals	--	--		132.22	228.86	
Level						***
(1) Medical center	--	--		70.65	122.21	(3)> (1),(2)
(2) Regional hospital	--	--		86.03	139.94	(2)> (1)
(3) Community hospital and clinics	--	--		126.62	222.56	

^1^ log transformation; S.D: standard deviation; *p < 0.05 **p < 0.01 ***p < 0.001

Regarding the utilization of family meetings (Table 3), we found that people of older age, female, marital status, poor, urban dwellers, and had cancer or major illness were positively associated with the use of family meetings. The results also revealed younger age and married were negatively associated with the initiation time of 1st family meeting, and poverty and medical condition were positively associated with the initiation time of 1st family meeting. The association between ownership and accreditation level of the site of the first family meeting held was also significant, the meetings held in the not-for-profit hospital were convened late, but held in private hospitals were earlier. Family meetings held in regional hospitals, community hospitals and clinics were earlier than medical centers.

**Table 3:** Factors associate with the utilization of family meeting

	Use of family meetings			Initiation time^1^		
	OR	LCL	UCL	ß	s.e	*p*-value
Age (reference = 66–85)						
≤ 6	0.559	0.420	0.744	0.295	0.194	0.1290
7–12	0.845	0.635	1.126	-0.152	0.190	0.4251
13–18	0.746	0.613	0.907	-0.331	0.132	0.0119
19–40	0.848	0.805	0.893	-0.184	0.034	< 0.0001
41–65	0.989	0.97	1.009	-0.107	0.013	< 0.0001
≥ 86	1.044	1.026	1.062	0.201	0.014	< 0.0001
Gender (reference = male)						
Female	1.044	1.026	1.062	-0.002	0.011	0.8503
Marital status (reference = single)						
Married	1.155	1.118	1.194	-0.047	0.022	0.0296
Divorced	1.128	1.084	1.175	-0.010	0.026	0.7099
Widowed	1.098	1.058	1.139	-0.002	0.024	0.9370
Poverty (reference = no)						
Yes	1.057	1.024	1.091	0.236	0.021	< 0.0001
Urbanization level(reference = rural)						
Urban	1.197	1.173	1.222	0.003	0.014	0.8413
Rural						
Medical condition (reference = none of cancer and major illnesses)						
Cancer	3.992	3.912	4.074	0.580	0.014	< 0.0001
Major illness	2.737	2.679	2.797	0.946	0.014	< 0.0001
Site of first family meeting (reference = public hospitals)						
Not-for-profit hospitals	--	--	--	-0.036	0.011	0.0014
Private Hospitals	--	--	--	0.049	0.020	0.0158
Level (reference = medical center)						
Regional hospital	--	--	--	0.121	0.012	< 0.0001
Community hospital and clinics	--	--	--	0.217	0.019	< 0.0001

^1^log transformation; OR: odds ratio; LCL: lower control limit; UCL: upper control limit; S.E.: standard error

Table 4 shows the effects of the utilization of family meetings on end-of-life care, the results demonstrate that the use of family meetings was positively associated with the use of hospice (OR = 4.949, 95% C.I.= 4.856–5.044) and negatively associated with the use of CPR (OR = 0.208, 95% C.I. =0.199–0.218) after adjusting demographic characteristics. The results also revealed that demographic characteristics affected the type of end-of-life care as well. But the effects were varied among demographic characteristics. The results also revealed the initiation time of the first family meeting was also associated with the type of end-of-life care. Compared to the reference group, while the first meeting was held 16–180 days before death, the opportunity for the use of hospice was 1.353–1.727 times higher, the OR was the highest when the first family meeting was held 31 to 45 day (OR = 1.727, 95%C.I.=1.630–1.831). However, if the first family meeting was held more than 180 days before death, the use of hospice care was less likely to implement (OR = 0.828, 95%C.I.= 0.781–0.877). In contrast, the effects of the initiation time of the first family meeting on CPR use were different from that on hospice use. If the family meeting is held 180 days before death, the opportunity for CPR use was significantly higher than reference group (OR = 1.187, 95%C.I.= 1.041–1.355), and the odds ratio of the use of *CPR* was lowest when the first family meeting was held 31 to 45 days before death (OR = 0.170, 95%C.I.= 0.136–0.213). Same, demographic characteristics and the characteristic of the site of first family meeting held were associated with type of end-of-life care, except for marital status, and the effects were also varied among demographic characteristics and the characteristic of the site of first family meeting held.

**Table 4:** Effects of family meetings on the end-of-life care

	Use of end-of-life care						Use of end-of-life care						
	HPC			CPR			HPC				CPR		
	OR	LCL	UCL	OR	LCL	UCL	OR	LCL	UCL	OR		LCL	UCL
Use of family meeting (reference = No)	4.949^***^	4.856	5.044	0.208^***^	0.199	0.218							
Initiation time of 1st family meeting (reference = < 15 days)													
16–30							1.612^***^	1.535	1.692	0.170^***^		0.136	0.213
31–45							1.727^***^	1.630	1.831	0.371^***^		0.302	0.457
46–60							1.698^***^	1.585	1.818	0.610^***^		0.496	0.751
61–90							1.469^***^	1.377	1.566	0.627^***^		0.518	0.760
91–180							1.353^***^	1.275	1.437	0.862		0.737	1.008
> 181							0.828^***^	0.781	0.877	1.187^*^		1.041	1.355
Age (reference = 66–85)													
≤ 6	0.932	0.762	1.140	3.782^***^	3.356	4.261	1.286	0.701	2.358	1.760		0.531	5.831
7–12	0.903	0.713	1.144	3.476^***^	2.943	4.104	2.337^*^	1.186	4.605	1.615		0.38	6.856
13–18	0.840^*^	0.714	0.988	4.799^***^	4.37	5.270	0.963	0.634	1.461	2.884^**^		1.348	6.173
19–40	1.015	0.973	1.058	2.265^***^	2.193	2.339	1.290^***^	1.153	1.443	1.268		0.949	1.694
41–65	1.280^***^	1.259	1.300	1.517^***^	1.491	1.543	1.271^***^	1.218	1.326	1.069		0.947	1.207
≥ 86	0.942^***^	0.924	0.961	0.519^***^	0.509	0.529	0.969	0.925	1.014	0.575^***^		0.502	0.659
Female (reference = male)	1.269^***^	1.251	1.288	0.989	0.975	1.004	1.156^***^	1.115	1.200	0.977		0.880	1.085
Marital status (reference = single)													
Married	1.073^***^	1.044	1.102	1.209^***^	1.179	1.24	1.022	0.950	1.098	1.008		0.828	1.227
Divorced	0.981	0.949	1.015	1.024	0.992	1.055	1.004	0.920	1.097	1.181		0.937	1.488
Widowed	0.934^***^	0.906	0.964	0.994	0.965	1.023	0.935	0.863	1.013	0.926		0.743	1.155
Poverty (reference = no)	0.859^***^	0.835	0.884	0.913^***^	0.889	0.938	0.829^***^	0.774	0.888	1.095		0.918	1.307
Rural (reference = urban)	1.559^***^	1.532	1.587	0.940^***^	0.925	0.954	1.048^*^	1.001	1.097	0.871^*^		0.767	0.988
Medical condition (reference = none of cancer and major illnesses)													
Cancer	19.004^***^	18.652	19.362	0.172^***^	0.168	0.176	5.395^***^	5.157	5.644	0.267^***^		0.235	0.304
Major illness	3.184^***^	3.118	3.252	0.485^***^	0.477	0.493	1.219^***^	1.162	1.279	0.550^***^		0.488	0.619
Site of first family meeting (reference = public hospitals)													
Not-for-profit hospitals							1.330^***^	1.283	1.379	0.867^**^		0.779	0.964
Private Hospitals							0.808^***^	0.755	0.865	1.488^***^		1.258	1.761
Level (reference = medical center)													
Regional hospital							0.897^***^	0.864	0.931	1.041		0.932	1.161
Community hospital and clinics							0.368^***^	0.346	0.391	0.739^**^		0.619	0.881

OR: odds ratio; LCL: lower control limit; UCL: upper control limit; S.E.: standard error; ^*^p < 0.05;^**^p < 0.01;^***^p < 0.001

## Discussion

In our study, we found that the utilization of family meetings had increased over time after the insurance reimbursement, and varied by demographic and healthcare organization characteristics. This study also found the use of family meetings positively associated with the use of hospice and negatively associated with the use of CPR, the initiation time was crucial for implementing hospice care and reducing the use of CPR.

Beyond these specific findings, there are several issues still worth further discussion. First of all, the variation in use of family meeting among demographic characteristics. Most of our findings were compatible with common sense, such as age and health status. Literacy [17] and family support [18] could also explain the differences we found between urban and rural residence and the differences by marital status. In addition, social values are important factors that could affect the use of family meetings by people with different demographic characteristics. In patriarchal societies, men are expected to be the primary wage-earners and support their family financially, and Taiwan is no exception. In Taiwan, despite decades of gender equality efforts, male preference still exists, but it has gradually diminished over the years of industrialization [19, 20]. Although the rights granted by law in Taiwan are now equal regardless of gender, the situation of male superiority has not completely disappeared. Therefore, at the moment of decision making, family members are less likely to accept the death of male family members and may continue to choose more aggressive treatment. Such cultural issues could help to explain the gender differences found in this study.

The effects of family meetings deserve further discussion and can be improved. In this study, we found that family meetings positively influenced hospice usage and reduced the likelihood of CPR being used. However, we also discovered that nearly half (46.64%) of those who participated in family meetings did not receive hospice care, and 2.53% still opted for CPR despite having had a family meeting. Previous research has indicated that the quality of these meetings is a crucial factor in their success [3, 10, 21]. Although there are many guidelines for implementing family meetings, most were developed in Western countries. Are these guidelines suitable for an Eastern society? Cultural issues should be considered. Furthermore, existing studies also found that a provider’s characteristics (e.g., gender, seniority, clinical specialty) [22, 23] and attitude [24, 25] toward end-of-life treatment affected the patient’s and family’s decision-making. Although these issues are related to family meeting quality, most of this information was unavailable to past studies. Future studies should examine the role of the quality of family meetings. Our findings also revealed that, in addition to family meetings, demographic characteristics and the nature of the site where the first meeting is held influence the type of end-of-life care provided. This suggests that the content of family meetings should be customized to address different demographic factors. Furthermore, it is important to investigate variations based on different accreditation levels and ownership types.

Thirdly, the optimum time to initiate the family meeting. As mentioned earlier, developing a plan of care is one of the purposes of a family meeting [11, 26]. Our data showed that there was about 50% of family meeting user initiated their first meeting in 30 days before death, the time of initiating in the first family meeting was close to the end of life. Recent studies on palliative care have shown that early initiation of palliative care helps to improve the patient’s quality of life [27]. How should the optimum time to initiate the family meeting be determined? Will the optimum time itself vary by demographic and health characteristics? Medical societies and health authorities should develop strategies to guide people who are approaching the end of life to convene a family meeting at the right time.

Last, In this study, we examined the use of family meetings, utilizing reimbursement codes to identify whether patients participated in these meetings. However, it is important to note that some family meetings may occur in clinical practice without being billed. In Taiwan, it is common for family members to accompany patients during hospitalization. This allows for opportunities to meet with attending physicians during ward rounds to discuss the patient’s condition and treatment preferences. Although these discussions may not meet the National Health Insurance Administration (NHIA) requirement of lasting longer than an hour, they can still be considered a form of family meeting. Consequently, the utilization of family meetings in this study may be underestimated.

### Limitations

Although this study is a nationwide, population-based investigation, some limitations still exist. Firstly, as mentioned earlier, there may have been unrecorded family meetings, which could lead to an underestimation of family meeting utilization. Secondly, some potentially relevant variables were not available for analysis. Previous literature has indicated some factors might impede end-of-life care discussion, e.g. culture [28], religious belief [29], patient or family knowledge and attitudes, and some were the facilitators, e.g. education attainment [30], organizational support. Studies also found patient-physician relationship and communication [31] also played an important role as well. In addition, patient’s clinical conditions (e.g. disease severity, pain index, and etc.) were not available, without these factors might diminish the validity of this study.

## Conclusion

In this study, we clearly demonstrated that family meetings affected the care at end-of-life with more hospice palliative care, and also less resuscitation at the end of life. However, there is still room for further more utilization and promotion of family meetings. Whist, health authorities and and health care providers should pay attention to the differences among various groups, and propose a comprehensive strategy for improving quality of life and good end-of-life care for all people.

## Data Availability

The data that support the findings of this study are available from Ministry of Health Welfare Taiwan but restrictions apply to the availability of these data, which were used under license for the current study, and so are not publicly available. Data are however available from the authors upon reasonable request and with permission of the Ministry of Health Welfare Taiwan. (https://dep.mohw.gov.tw/dos/cp-2516-59203-113.html)
